# Prognostic Value of Lymph Node Dissection for Intrahepatic Cholangiocarcinoma Patients With Clinically Negative Lymph Node Metastasis: A Multi-Center Study From China

**DOI:** 10.3389/fonc.2021.585808

**Published:** 2021-03-11

**Authors:** Qiao Ke, Lei Wang, Ziguo Lin, Jianying Lou, Shuguo Zheng, Xinyu Bi, Jianming Wang, Wei Guo, Fuyu Li, Jian Wang, Yamin Zheng, Jingdong Li, Shi Cheng, Weiping Zhou, Yongyi Zeng

**Affiliations:** ^1^Department of Hepatobiliary Surgery, Mengchao Hepatobiliary Hospital of Fujian Medical University, Fuzhou, China; ^2^Department of Radiation Oncology, Fujian Cancer Hospital, The School of Clinical Medicine, Fujian Medical University, Fuzhou, China; ^3^Department of Hepatobiliary Surgery, The Second Hospital Affiliated to Zhejiang University, Hangzhou, China; ^4^Department of Hepatobiliary Surgery, The Southwest Hospital Affiliated to the Army Medical University, Chongqing, China; ^5^Department of Hepatobiliary Surgery, Cancer Hospital, Chinese Academy of Medical Sciences, Beijing, China; ^6^Department of Hepatobiliary Surgery, Tongji Hospital Affiliated to Tongji Medical College, Huazhong University of Science & Technology, Wuhan, China; ^7^Department of Hepatobiliary Surgery, Beijing Friendship Hospital Affiliated to Capital Medical University, Beijing, China; ^8^Department of Hepatobiliary Surgery, The West China Hospital of Sichuan University, Chengdu, China; ^9^Department of Hepatobiliary Surgery, Renji Hospital Affiliated to Shanghai Jiaotong University, Shanghai, China; ^10^Department of Hepatobiliary Surgery, Xuanwu Hospital Affiliated to Capital Medical University, Beijing, China; ^11^Department of Hepatobiliary Surgery, The Affiliated Hospital of Chuanbei Medical University, Nanchong, China; ^12^Department of Hepatobiliary Surgery, Tiantan Hospital Affiliated to Capital Medical University, Beijing, China; ^13^Department of Hepatobiliary Surgery III, Eastern Hepatobiliary Surgery Hospital, Secondary Military Medical University, Shanghai, China

**Keywords:** intrahepatic cholangiocarcinoma, lymph node dissection, node-negative, overall survival, propensity score matching

## Abstract

**Background:**

The clinical value of lymph-node dissection (LND) for intrahepatic carcinoma (ICC) patients with clinically negative lymph node metastasis (LNM) remains unclear; hence we conducted a multi-center study to explore it.

**Methods:**

Patients who were diagnosed ICC with clinically negative LNM and underwent hepatectomy with or without LND from December 2012 to December 2015 were retrospectively collected from 12 hepatobiliary centers in China. Overall survival (OS) was analyzed using the Kaplan–Meier method, and then subgroup analysis was conducted stratified by variables related to the prognosis.

**Results:**

A total of 380 patients were eligible including 106 (27.9%) in the LND group and 274 (72.1%) in the non-LND group. Median OS in the LND group was slightly longer than that in the non-LND group (24.0 *vs.* 18.0 months, *P* = 0.30), but a significant difference was observed between the two groups (24.0 *vs.* 14.0 months, *P* = 0.02) after a well-designed 1:1 propensity score matching without increased severe complications. And, LND was identified to be one of the independent risk factors of OS (HR = 0.66, 95%CI = 0.46–0.95, *P* = 0.025). Subgroup analysis in the matched cohort showed that patients could benefit more from LND if they were male, age <60 years, had no HBV infection, with ECOG score <2, CEA ≤5 ug/L, blood loss ≤400 ml, transfusion, major hepatectomy, resection margin ≥1 cm, tumor size >5 cm, single tumor, mass-forming, no satellite, no MVI, and no perineural invasion (all *P* < 0.05). Furthermore, only patients with pathologically confirmed positive LNM were found to benefit from postoperative adjuvant therapy (*P* < 0.001).

**Conclusion:**

With the current data, we concluded that LND would benefit the selected ICC patients with clinically negative LNM and might guide the postoperative management.

## Introduction

The incidence of intrahepatic cholangiocarcinoma (ICC) has been increasing in recent decades worldwide (1, 2). The long-term outcome of ICC patients receiving surgical resection remains far from satisfactory (3, 4) partly because of the unique lymph node metastasis (LNM) with an incidence of 30 to 40% (5, 6). To conduct lymph node dissection (LND) for ICC patients with clinically positive LNM has gained a consensus, but it remains controversial for patients with clinically negative LNM. The reasons are as follows: 1) survival benefit has not been confirmed yet in a recent meta-analysis (7), and 2) increasing risk of complications related to LND stirs up the surgeons’ hesitation (8, 9).

However, considering that the preoperative imaging is far from enough to ensure an accurate N staging and the postoperative management of ICC is poor, LND has been tried in more and more hepatobiliary centers. Hu et al (10). firstly reported that routine LND would not improve the prognosis of ICC patients with clinically negative LNM in a retrospective study from single center, but the survival benefit was well identified by a recent multi-center study derived from France and Japan (11), which shed light on routine LND for ICC patients with clinically negative LNM.

As is known to all, the incidence of ICC is higher in China than in most part of the world (12). Hence, we want to verify the clinical value of LND for ICC patients with clinically negative LNM using the data from a multi-center study in China and identify the potential beneficiary of regional LND.

## Material and Methods

This study was conducted under the ethical guideline of the 1975 Declaration of Helsinki and was approved by Mengchao Hepatobiliary Hospital of Fujian Medical University’s Ethics Committee (No. 2018_048_01). Individual informed consent was waived by the ethics committee mainly because patient medical data including clinicopathological information and follow-up were extracted retrospectively. Data of patients receiving surgical resection between December 2012 and December 2015 were collected from multi-centers in China, including Mengchao Hepatobiliary Hospital of Fujian Medical University, Eastern Hepatobiliary Surgery Hospital, Affiliated Cancer Hospital of Chinese Academy of Medical Sciences, Tongji Hospital Affiliated to Tongji Medical College, Huazhong University of Science & Technology, Beijing Friendship Hospital Affiliated to Capital Medical University, Xuanwu Hospital Affiliated to Capital Medical University, Tiantan Hospital Affiliated to Capital Medical University, the Affiliated Hospital of Chuanbei Medical University, Renji Hospital Affiliated to Shanghai Jiaotong University, the West China Hospital of Sichuan University, the Southwest Hospital Affiliated to the Army Medical University, and the Second Hospital of Zhejiang University.

### Eligibility

Patients were enrolled in this study if they 1) had been pathologically diagnosed as ICC, 2) had no LNM identified by preoperative computed tomography (CT)/magnetic resonance imaging (MRI), and 3) underwent an R0 resection with or without regional LND. Patients who had 1) preoperative obstructive jaundice, 2) extrahepatic metastasis, 3) received preoperative adjuvant treatments, and 4) died within one month following resection were excluded in this study.

### Interventions

Generally, surgical procedure included hepatectomy with or without LND. Hepatectomy was conducted for patients who met the following criteria: 1) technically resectable tumor with no evidence of extrahepatic metastasis, 2) good general condition, well-tolerated liver function, and sufficient residual liver volume.

LND was conducted on condition that: 1) patient was considered to be most likely with LNM by a multiple discipline team discussion before operation, 2) swollen lymph nodes were detected manually by surgeons in the surgical procedure. LND in this study generally referred to regional LND. The procedure of LND included skeletonization of hepatoduodenal ligament and resection of para hepatic artery lymph nodes at least to the second station, which was a little different from each center.

Adjuvant treatment (AT) including transarterial chemoembolization (TACE), chemotherapy, radiotherapy, or chemoradiotherapy was conducted one month after radical resection to reduce recurrence and improve prognosis. Briefly, the preferred regimen was one or two courses of TACE, 4–6 courses of fluoropyrimide- or gemcitabine-based chemotherapy regimens, and intensity-modulated radiation therapy with a total dose of 45–50 Gy at 1.8–2.0 Gy/fractions, which was a little different from each center.

### Definition

Clinically negative LNM was defined as patients without any suspicious or positive LNM determined by contrast CT or MRI (13, 14), and clinically positive LNM was defined as lymph node with short axis diameter >10 mm, central necrosis, and inhomogeneous enhancement (14, 15).

Major hepatectomy was defined as resection of three or more liver segments according to the Couinaud’s classification, while resection of less than three segments was defined as minor hepatectomy (16).

Radical resection was defined as no tumor residual at surgical margin under microscopy (17). Wide surgical margin was defined as the distance from the surgical margin to tumor edge exceeding 1 cm, while narrow surgical margin was the distance within 1 cm (18).

Histopathological specimens were evaluated by three independent pathologists. The morphological status was grouped as mass-forming (MF), intraductal growth (IG), and periductal infiltrating (PI) (3), and the differentiation was graded as well, moderate and poor.

Patients receiving LND was divided into negative pLNM (without pathologically confirmed LNM) and positive pLNM (with at least one pathologically confirmed LNM), while patients without LND were defined as non-LND.

Perineural invasion was defined as the presence of tumor cells around the nerve (19), and microvascular invasion (MVI) was defined as a portal vein, hepatic vein, or large capsule vessel of the liver tissue adjacent to the tumor edge (20).

Chemoradiotherapy was defined as patients receiving both adjuvant chemotherapy and adjuvant radiotherapy, regardless of whether sequential or simultaneous.

Adverse events (AEs) related to surgery were evaluated according to the Clavien-Dindo classification (21), and Grade III and above were defined as severe AEs.

### Follow-Up and Definition of Endpoints

All patients were periodically followed up once every 2–3 months in the first 2 years and then once every 6 months. Routine follow-up items included liver function tests, serum levels of CA19-9, and CEA, and abdominal ultrasound, and a contrast-enhanced CT or MRI was warranted once recurrence was clinically suspected. Recurrence or metastasis was defined as new lesions with radiologic characteristics of ICC (22), and further treatment was immediately adopted whenever recurrence was confirmed.

The primary endpoint was overall survival (OS), which was calculated from the data of resection to either the data of death or the latest follow-up. The secondary endpoint was AE related to surgery including operation time, blood loss, transfusion, and hospital stay, which were extracted from the medical records.

### Clinicopathological Variables

Potential variables associated with the prognosis of HCC patients were determined according to previous studies (23). Tumor diameter (<5 cm *vs.* ≥5 cm) and tumor number (single *vs.* multiple) were categorized according to the American Joint of Cancer Committee (AJCC) system (23). HBV infection was defined as history of HBV infection, regardless of status of HBsAg and HBV-DNA (23). The data of blood transfusion was extracted from anesthesia records, which typically included intraoperative transfusion of red blood cell and plasma (24). Age (<60 years *vs.* ≥60 years), blood loss (≤400 *vs.* >400 ml), surgical margin (wide *vs.* narrow), MF (no *vs.* yes), and differentiation (well-moderate *vs.* poor) were categorized as previous studies reported (23), and preoperative levels of CA19-9 (<37 *vs.* ≥37 U/ml) and CEA (<5 *vs.* ≥5 ng/ml) were categorized using cutoff value provided by clinical references (10).

### Statistics

Propensity score matching (PSM) was adopted to minify the selection bias (25). Variables such as sex, age, underlying liver disease, ECOG score, and tumor characteristics including size and number were used to be matched using a 1:1 nearest neighbor method with a caliber of 0.20.

Considering that continuous variables in this study were re-defined as categorical variables, all the variables were evaluated by the chi-square test or Fisher’s exact test between the two groups before and after PSM. The clinical efficacy of LND and AT was determined by the Kaplan–Meier method, and medians with hazard ratio and confidence interference (CI) 95% were evaluated using log-rank test. Prognostic factors with *P <*0.05 using the log-rank test were then enrolled into multivariable Cox proportional hazards model to identify potential independent risk factors. Of note, variate of LND was abandoned due to collinearity might exist between variates of LND and LNM.

Subgroup analyses of sex (female *vs.* male), age (≤60 *vs.*>60 years), HBV infection (no *vs.* yes), the Eastern Cooperative Oncology Group (ECOG) score (<2 *vs.* ≥2),CA19-9 (≤37 *vs.* >37 U/mL), CEA (≤5 *vs.* >5 µg/L), blood loss (≤400 *vs.* >400 ml), resection margin (≥1 *vs.* <1 cm), tumor diameter (≤5 *vs.* >5 cm), tumor number (single *vs.* multiple), MF (no *vs.* yes), tumor differentiation (well-moderate *vs.* poor), satellite (no *vs.* yes), MVI (no *vs.* yes), and perineural invasion (no *vs.* yes) for OS were performed using log-rank test before and after PSM, and then forest plot of subgroup analysis was depicted with each estimated HR and 95% CI.

All statistical analyses were conducted using Rstudio 3.6.1 including packages of “table1”, “MatchIt”, “survminer”, “survival”, “plyr”, “forestplot”. All P values were two sided, and *P <*0.05 was considered statistically significant.

## Results

**Figure 1:**
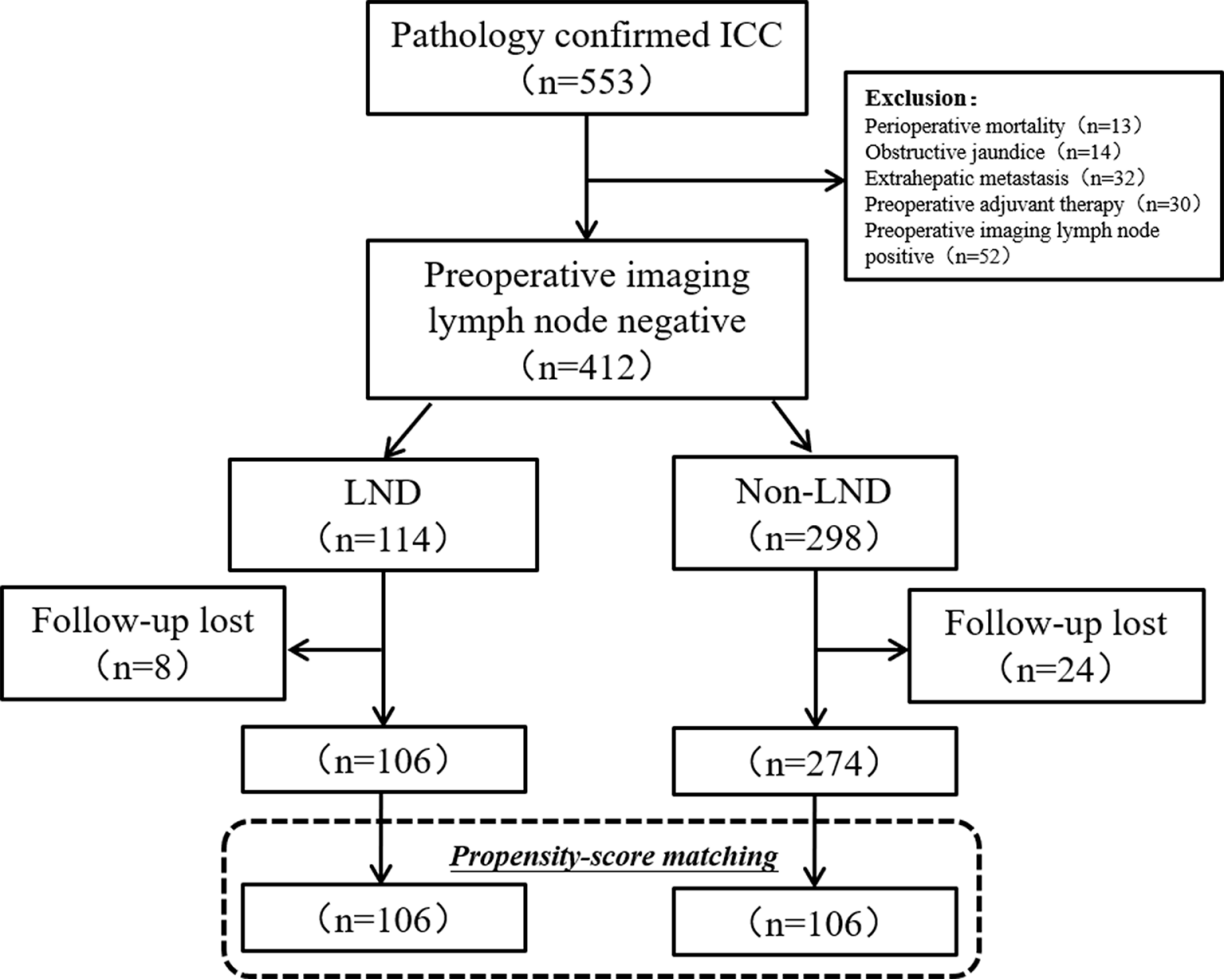
Flow chart of patients’ enrollment. ICC, intrahepatic carcinoma; LND, lymph node dissection.

Initially, 553 patients with ICC underwent resection, but 14 patients (2.5%) were excluded for preoperative obstructive jaundice, 13 (2.3%) were for death within one month, 32 (5.7%) were for extrahepatic metastasis, and 52 (9.4%) were for the preoperative imaging lymph node positive. During the period of follow-up (1–66 months), 32 patients (7.8%) lost to follow-up. In the final, 380 patients with clinically node-negative remained to be analyzed, including 106 (27.9%) in the LND group and 274 (72.1%) in the non-LND group (**Figure 1**).

### Patients’ Characteristics

Baseline characteristics of patients were summarized in **Table 1** before and after PSM. In the crude cohort, the proportion of female patients, CA19-9 >37 U/ml, CEA >5 µg/L, narrow margin (≤1 cm), and perineural invasion in the LND group was higher than that in the non-LND group (all *P* < 0.05), but the proportion of HBV infection in the LND group was lower than that in the non-LND group (*P* = 0.004). However, no significant differences were observed in all the variables after PSM (all *P* > 0.05).

**Table 1:** Clinicopathological characteristics before and after PSM.

		Before PSM			After PSM		
		Non-LND	LND	*P*-Value	Non-LND	LND	*P*-Value
		(n = 274)	(n = 106)		(n = 106)	(n = 106)	
**Sex**	Female	89 (32.5%)	52 (49.1%)	0.004	50 (47.2%)	52 (49.1%)	0.891
	Male	185 (67.5%)	54 (50.9%)		56 (52.8%)	54 (50.9%)	
**Age**	<60years	161 (58.8%)	54 (50.9%)	0.207	49 (46.2%)	54 (50.9%)	0.583
	≥60 years	113 (41.2%)	52 (49.1%)		57 (53.8%)	52 (49.1%)	
**HBV**	No	170 (62.0%)	83 (78.3%)	0.004	81 (76.4%)	83 (78.3%)	0.870
	Yes	104 (38.0%)	23 (21.7%)		25 (23.6%)	23 (21.7%)	
**ECOG score**	<2	232 (84.7%)	91 (85.8%)	0.898	86 (81.1%)	91 (85.8%)	0.459
	≥2	42 (15.3%)	15 (14.2%)		20 (18.9%)	15 (14.2%)	
**CA19-9**	≤37 U/ml	198 (72.3%)	56 (52.8%)	<0.001	58 (54.7%)	56 (52.8%)	0.89
	>37 U/ml	76 (27.7%)	50 (47.2%)		48 (45.3%)	50 (47.2%)	
**CEA**	≤5 µg/L	223 (81.4%)	75 (70.8%)	0.034	70 (66.0%)	75 (70.8%)	0.555
	>5 µg/L	51 (18.6%)	31 (29.2%)		36 (34.0%)	31 (29.2%	
**Major hepatectomy**	No	84 (30.7%)	28 (26.4%)	0.491	28 (26.4%)	28 (26.4%)	1
	Yes	190 (69.3%)	78 (73.6%)		78 (73.6%)	78 (73.6%)	
**Resection margin**	≥1 cm	185 (67.5%)	59 (55.7%)	0.041	64 (60.4%)	59 (55.7%)	0.578
	<1 cm	89 (32.5%)	47 (44.3%)		42 (39.6%)	47 (44.3%)	
**Tumor diameter**	≤5 cm	105 (38.3%)	34 (32.1%)	0.310	33 (31.1%)	34 (32.1%)	1.000
	>5 cm	169 (61.7%)	72 (67.9%)		73 (68.9%)	72 (67.9%)	
**Tumor Number**	Single	190 (69.3%)	79 (74.5%)	0.384	73 (68.9%)	79 (74.5%)	0.446
	Multiple	84 (30.7%)	27 (25.5%)		33 (31.1%)	27 (25.5%)	
**Mass-forming**	No	97 (35.4%)	34 (32.1%)	0.623	34 (32.1%)	34 (32.1%)	1.000
	Yes	177 (64.6%)	72 (67.9%)		72 (67.9%)	72 (67.9%)	
**Differentiation**	Well &Moderate	223 (81.4%)	82 (77.4%)	0.459	87 (82.1%)	82 (77.4%)	0.494
	Poor	51 (18.6%)	24 (22.6%)		19 (17.9%)	24 (22.6%)	
**Satellite**	No	197 (71.9%)	80 (75.5%)	0.566	74 (69.8%)	80 (75.5%)	0.441
	Yes	77 (28.1%)	26 (24.5%)		32 (30.2%)	26 (24.5%)	
**MVI**	No	251 (91.6%)	96 (90.6%)	0.905	98 (92.5%)	96 (90.6%)	0.805
	Yes	23 (8.4%)	10 (9.4%)		8 (7.5%)	10 (9.4%)	
**Perineural invasion**	No	256 (93.4%)	89 (84.0%)	0.008	94 (88.7%)	89 (84.0%)	0.424
	Yes	18 (6.6%)	17 (16.0%)		12 (11.3%)	17 (16.0%)	

LND, lymph node dissection; PSM, propensity score matching; HBV, hepatitis B virus; ECOG, the Eastern Cooperative Oncology Group; MVI, microvascular invasion.

### Operative and Postoperative Outcomes

The median number of lymph node dissection was 3.5 (1–39). The proportions of operation time >180 min, hospital stay >15 days, blood loss >400 ml, transfusion, and the total AE related to the surgery in the LND group were significantly higher than those in the non-LND group (all *P* < 0.05, **Table 2**) before and after PSM, but no significant differences were observed in the incidences of severe AEs between the two groups before and after PSM (both *P* > 0.05). Of note, the proportion of patients receiving p-AT in the LND group was slightly higher than that in the non-LND group (27.4 *vs.* 18.2%, *P* = 0.069) before PSM, but they were comparable in the matched cohort (27.4 *vs.* 23.6%, *P* = 0.636).

**Table 2:** Operative and postoperative outcomes before and after PSM.

		Before PSM			After PSM		
		Non-LND	LND	*P*-Value	Non-LND	LND	*P*-Value
		(n = 274)	(n = 106)		(n = 106)	(n = 106)	
**Operation Time**	≤180min	172 (62.8%)	36 (34.0%)	<0.001	61 (57.5%)	36 (34.0%)	<0.001
	>180min	102 (37.2%)	70 (66.0%)		45 (42.5%)	70 (66.0%)	
**Hospital stay**	≤15days	249 (90.9%)	77 (72.6%)	<0.001	95 (89.6%)	77 (72.6%)	0.003
	>15days	25 (9.1%)	29 (27.4%)		11 (10.4%)	29 (27.4%)	
**Blood loss**	≤400 mL	264 (96.4%)	79 (74.5%)	<0.001	101 (95.3%)	79 (74.5%)	<0.001
	>400 mL	10 (3.6%)	27 (25.5%)		5 (4.7%)	27 (25.5%)	
**Transfusion**	No	235 (85.8%)	71 (67.0%)	<0.001	86 (81.1%)	71 (67.0%)	0.019
	Yes	39 (14.2%)	35 (33.0%)		20 (18.9%)	35 (33.0%)	
**AE**	No	233 (85.0%)	74 (69.8%)	0.001	88 (83.0%)	74 (69.8%)	0.036
	Yes	41 (15.0%)	32 (30.2%)		18 (17.0%)	32 (30.2%)	
**severe AE**	No	248 (90.5%)	92 (86.8%)	0.289	97 (91.5%)	92 (86.8%)	0.270
	Yes	26 (9.5%)	14 (13.2%)		9 (8.5%)	14 (13.2%)	
**p-AT**	No	224 (81.8%)	77 (72.6%)	0.069	81 (76.4%)	77 (72.6%)	0.636
	Yes	50 (18.2%)	29 (27.4%)		25 (23.6%)	29 (27.4%)	

LND, lymph node dissection; PSM, propensity score matching; AE, adverse event; p-AT, postoperative adjuvant treatment.

### Long-Term Outcomes

**Figure 2:**
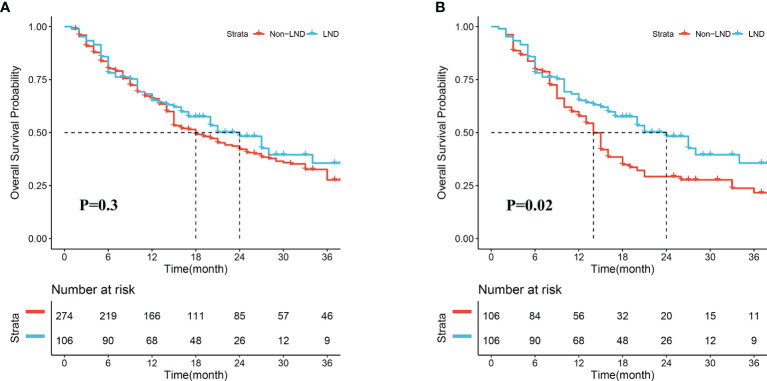
Overall survival of patients receiving lymph node dissection (LND) or not before **(A)** and after propensity score matching **(B)**.

In the crude cohort, median OS in the LND group was slightly longer than that in the non-LND group (24.0 *vs.* 18.0 months, *P* = 0.30, **Figure 2A**), and the 1-, 2-, and 3-year survival rates in the two groups were 66.0 *vs.* 67.5%, 53.8 *vs.* 48.5%, 50.0 *vs.* 40.1%, respectively. However, a significant difference was observed in the term of median OS between the two groups after PSM (24.0 *vs.* 14.0 months, *P* = 0.02, **Figure 2B**), and the 1-, 2-, and 3-year survival rates in the two groups were 66.0 *vs.* 60.4%, 53.8 *vs.* 37.7%, 50.0 *vs.* 34.0%, respectively.

### Univariate and Multivariate Analyses of Prognosis Factors Associated With Overall Survival

In the matched cohort, preoperative levels of CEA, transfusion, resection margin, LND, tumor number, tumor differentiation, satellite, and perineural invasion were identified to be associated with OS (all *P* < 0.05, **Table 3**) using univariate analysis. And then, LND (HR = 0.66, 95%CI = 0.46–0.95, *P* = 0.025), resection margin (HR = 0.65, 95%CI = 0.42–0.98, *P* = 0.048), and satellite (HR = 2.35, 95%CI = 1.32–4.20, *P* = 0.004) were independent risk factors for OS using multivariate analysis. Details were depicted in **Table 3**.

**Table 3:** Univariate and multivariate analysis of OS in patients with ICC after PSM.

	Univariate analysis			Multivariate analysis		
	HR	95%CI	*P*	HR	95%CI	*P*
**Sex (Female *vs.* Male)**	1.25	0.88–1.79	0.214			
**Age (<60 years *vs.* ≥60 years)**	0.85	0.6–1.21	0.373			
**HBV (No *vs.* Yes)**	1.22	0.8–1.85	0.35			
**ECOG score (<2 *vs.* ≥2 )**	1.24	0.79–1.94	0.346			
**CA19-9 (≤37 *vs.* >37 U/ml)**	1.03	0.72–1.46	0.884			
**CEA (≤5 *vs.* >5 µg/L)**	0.66	0.44–0.98	0.041			
**Blood loss (≤400 *vs.* >400 mL)**	0.59	0.24–1.44	0.244			
** Transfusion (No *vs.* Yes)**	0.59	0.36–0.97	0.039			
**Major hepatectomy (No *vs.* Yes)**	1.5	0.98–2.3	0.064			
**Resection margin (≥1 *vs.* <1 cm)**	0.51	0.35–0.75	0.001	0.65	0.42-0.98	0.048
**LND (No *vs.* Yes)**	0.64	0.45–0.92	0.016	0.66	0.46-0.95	0.025
**Tumor diameter (≤5 *vs.* >5 cm)**	1.45	0.97–2.17	0.067			
**Tumor number (Single *vs.* Multiple)**	1.55	1.06–2.27	0.024			
**Mass-forming (No *vs.* Yes)**	0.89	0.61–1.29	0.537			
**Differentiation (Well &Moderate *vs.* Poor)**	0.58	0.35–0.94	0.028			
**Satellite (No *vs.* Yes)**	2.07	1.43–3.02	<0.001	2.35	1.32-4.20	0.004
**MVI (No *vs.* Yes)**	1.32	0.74–2.35	0.344			
**Perineural invasion (No *vs.* Yes)**	0.54	0.3–0.97	0.041			
**p-AT (No *vs.* Yes)**	0.67	0.43–1.05	0.08			

LND, lymph node dissection; PSM, propensity score matching; HBV, hepatitis B virus; ECOG, the Eastern Cooperative Oncology Group; MVI, microvascular invasion; p-AT, postoperative adjuvant treatment.

### Subgroup Analysis of Overall Survival Stratified by Risk Factors

**Figure 3:**
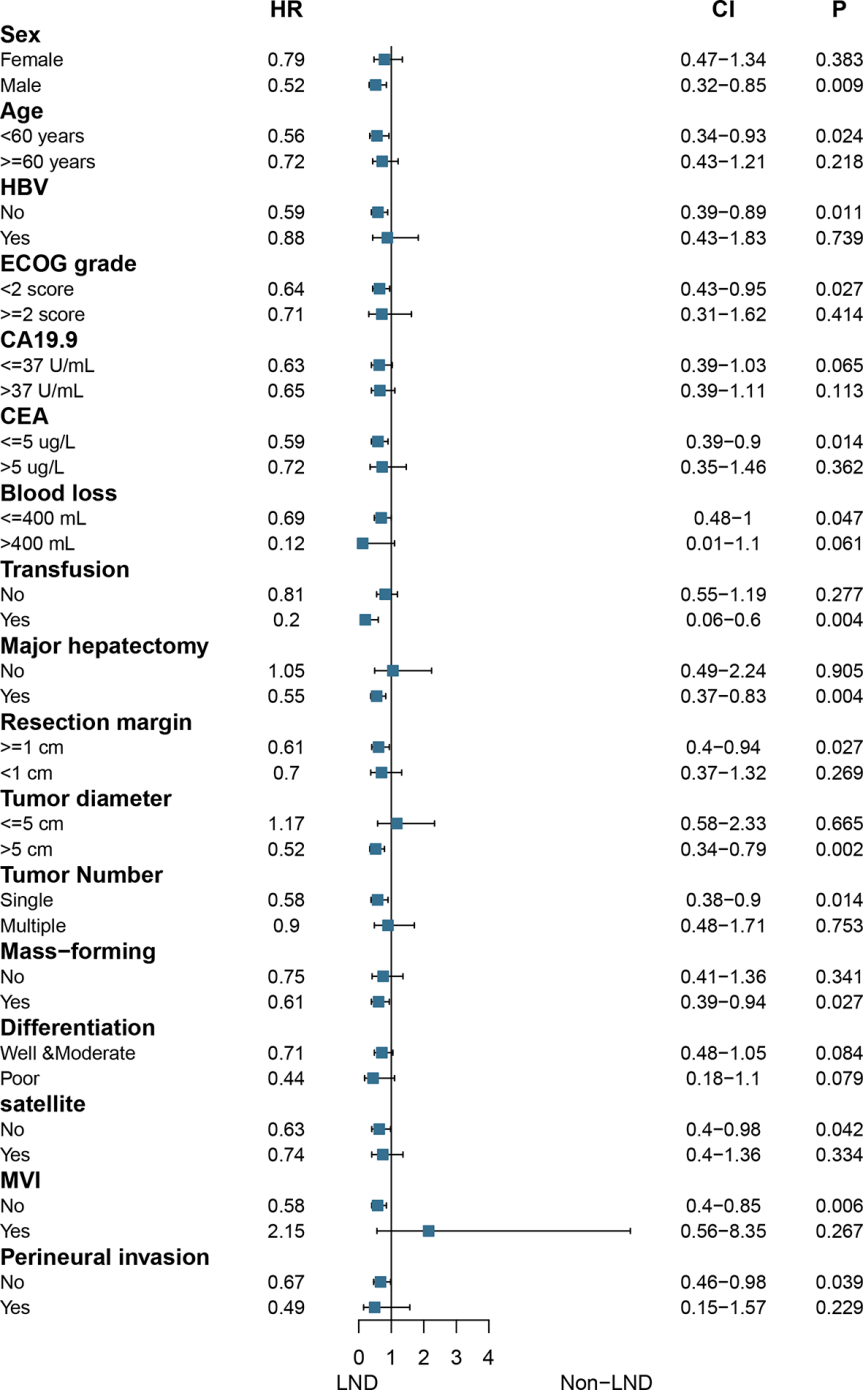
Forest plot of subgroup analysis stratified by risk factors in the matched cohort.

In the matched cohort, subgroup analysis showed that only patients with the following characteristics would benefit from LND: male, age <60 years, no HBV infection, ECOG score <2, CEA ≤5 ug/L, blood loss ≤400 ml, transfusion, major hepatectomy, resection margin ≥1 cm, tumor size >5 cm, single tumor, MF, no satellite, no MVI, and no perineural invasion (all *P* < 0.05, **Figure 3**).

### Effects of Lymph Node Dissection on the Postoperative Management of Intrahepatic Carcinoma

**Figure 4:**
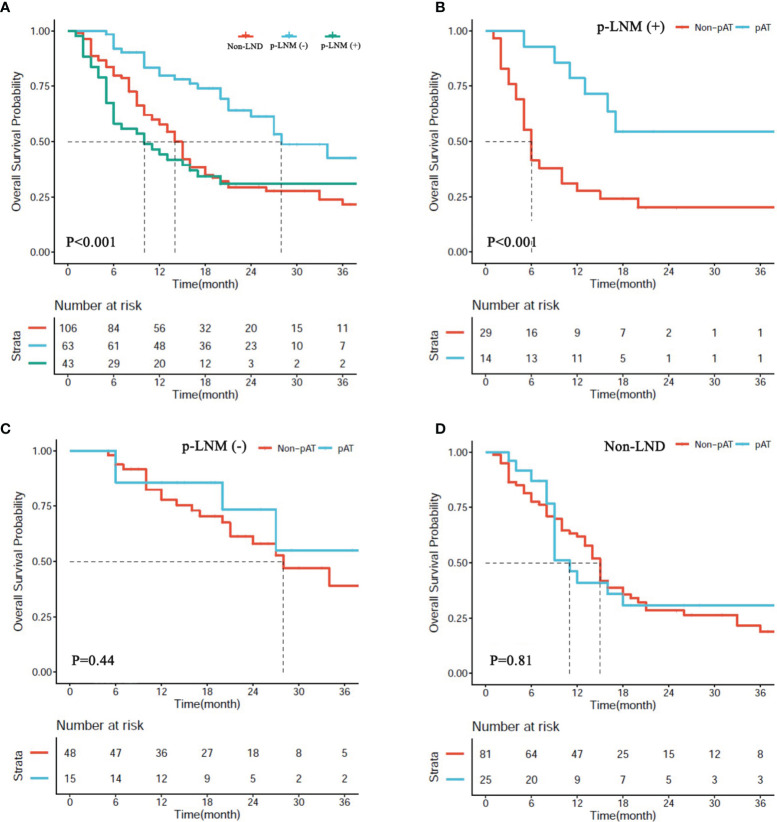
**(A)** Overall survival (OS) of patients with hepatectomy alone (non-LND), lymph node metastasis (p-LNM+), and no lymph node metastasis (p-LNM−). **(B–D)** OS of patients with p-LNM+, p-LNM−, and non-LND receiving postoperative adjuvant treatment or not.

In the matched cohort, 43 patients receiving LND were present with positive pLNM, 63 receiving LND were negative pLNM, and 106 receiving hepatectomy only were non-LND. Median OS of patients with negative pLNM was significantly longer than those in the patients with positive pLNM, and non-LND (28.0 *vs.* 10.0 months, *P* < 0.001; 28.0 *vs.* 14.0 months, *P* < 0.001; respectively, **Figure 4A**), and no significant difference was observed between patients with positive pLNM and non-LND (*P* > 0.05, **Figure 4A**).

In the matched cohort, 54 patients (25.5%) received AT following resection including 14 patients (32.6%) in the subgroup of positive pLNM, 15 (23.8%) in the subgroup of negative pLNM, and 25 (23.6%) in the subgroup of non-LND. The benefit of AT was observed in the subgroup of positive pLNM (*P* < 0.001, **Figure 4B**), but not in the subgroups of negative pLNM and non-LND (*P* = 0.81, **Figure 4C**; *P* = 0.41, **Figure 4D**; respectively).

## Discussion

LND is a precondition of LNM diagnosed by pathology (3), which is one of the most prominent risk factors associated with recurrence and poor prognosis in ICC patients following surgical resection (26, 27), but its efficacy for patients with clinically node-negative remains controversial. In this study, the clinical value of LND for patients with clinically node-negative was evaluated using a large-scale, well-matched cohort of patients derived from 12 highly experienced hepatobiliary centers in China. Results showed that LND was associated with a prolonged median OS of ICC patients with clinically node-negative, which was accordant with the recent study from France and Japan (11).

A high incidence of LNM is the unique characteristic of ICC (5, 6), but routine LND has not been recommended in the guidelines or expert consensus on ICC. In fact, the proportion of patients who received LND is considerably low (8, 28); the reasons might be as follows: 1) previous studies found that LND did not bring the benefit for ICC patients (7); and 2) LND was generally along with prolonged surgery time and increased surgical risk (7, 8). In addition, LND is conducted in highly experienced hepatobiliary centers, and the extent of LND was far from standardization, which is determined by each surgeon. Therefore, LND was not routinely conducted in the hepatectomy for ICC patients.

Preoperative lymph node staging is the determining factor for LND or not, but the current clinical lymph node staging is unsatisfactory. The sensitivity and specificity of contrast CT are reported to be 35.0–50.0% and 77.0–92.0% (29, 30), while as for MRI, they are 34.0–78.2% and 37.4–74.5% (31, 32). In this study, 43 of 106 patients (40.6%) with clinically negative LNM receiving LND were confirmed to be present with LNM by postoperative pathology, which indicated that preoperative imaging alone could not be enough to decide whether LND should be conducted for patients with ICC, especially for those with clinically negative LNM.

However, results from the only two published studies were apparently different from each other (10, 11). In the current multi-center study, LND was found to prolong the median OS of patients with clinically node-negative in a well-designed cohort (*P* < 0.05), which was coincident with the previous multi-center study. Further analysis showed that patients would be much more likely to benefit from LND if they were male, age <60 years, had no HBV infection, with ECOG score <2, CEA ≤5 ug/L, major hepatectomy, wide resection margin, MF, single tumor, tumor size >5 cm, no satellite, no perineural invasion, and no MVI (all *P* < 0.05), which indicated that not all patients will benefit from LND. Hence, we concluded that LND should be conducted in selected patients with clinically negative LNM.

The role of LND for node-negative ICC is not just to ensure an accurate N staging. In this study, LND was found to bring survival benefit for node-negative ICC in a matched cohort, and patients receiving LND are much more likely to receive p-AT (LND *vs.* non-LND: 27.4 *vs.* 18.2%, *P* = 0.069). Further analysis showed that positive pLNM patients suffered worse prognosis compared with those with negative pLNM and non-LND (*P* < 0.05), but only patients with positive LNM were found to benefit from postoperative adjuvant treatments (*P* < 0.05), which indicated that LND might also play an important role in the postoperative management of ICC.

Of note, we performed further analysis not according to the N staging but based on the pathological lymph node status in this study. Firstly, at least six lymph nodes should be harvested according to the 8^th^ AJCC guidelines (3), but the median harvested nodes were four (2–8) with the proportion of harvested lymph nodes exceeding six of 12.5% in the previous multi-center study (33). As for ICC with clinically negative LNM, it is considered to be fewer. In the current study, the median harvested nodes were 3.5 (1–39). Among the 106 patients who received LND, 30 patients (28.3%) with harvested lymph nodes exceeding six, and only 15 patients (14.2%) were diagnosed as N1. But among the remaining 76 patients (71.7%) with harvested lymph nodes <6, there were 28 patients (26.4%) who were found to have pathological LNM. In our opinion, it is acceptable that postoperative treatment could be guided according to the pathological lymph node status rather than N staging.

The optimal p-AT for resected ICC patients has not been determined, and it lacks clinical trial data to support a standard regimen in the postoperative management. Using the data from the multi-center study, we established a nomogram model (23), and found that only patients with “middle risk” could benefit from p-AT, which was confirmed by our recent meta-analysis based on the retrospective studies (34). Currently, adjuvant capecitabine chemotherapy with a duration of 6 months is recommended for patients with resected biliary tract cancer by ASCO guideline (35); while the 8^th^ NCCN guideline suggests observation or systemic therapy for ICC patients with R0 resection and fluoropyrimidine-/gemcitabine-based chemotherapy for patients with R1 or pLNM (3). In our previous multi-center study (23), a total of 77 patients received postoperative adjuvant treatment, including 32 (41.6%) patients received TACE, 21 (27.3%) received chemotherapy, 10 (13.0%) received radiotherapy, and 14 (18.2%) received chemoradiotherapy, but only postoperative chemotherapy and TACE were found to benefit ICC patients (both *P* < 0.05). However, in the era of novel chemotherapy, targeted drug like apatinib, immune checkpoint inhibitors like pembrolizumab, and intensity modulated radiation therapy (35, 36), the postoperative management of ICC with positive pLNM is very promising.

There were several limitations in this study. Firstly, selection bias and recalling bias were hard to avoid in a retrospective study, although a well-designed PSM was conducted. Imaging modalities, perioperative management, and surgical procedures varied from each center, and detailed data on chemotherapy drugs and dose, radiotherapy modality and dosage, and treatment after recurrence were missing. Second, it seems insufficient to make a decision on LND based on the current CT or MRI. In the current study, the false negative rate of LNM was as high as 40.6%. Hence, PET-CT or laparoscopic exploration is strongly recommended if possible. The third limitation was the extension of LND as previously reported. Considering that the extension of LND was mostly determined by each surgeon and most of the data on the detailed extension of LND were hard to collect, we collected the number of harvested lymph nodes from the pathology reports. Even so, the median harvested lymph nodes of 3.5 were far from six to conduct N staging according to the 8^th^ AJCC guideline. In future, a prospective multi-center trial should be conducted, in which the extension of LND and the least harvested number should be well designed and implemented.

## Conclusion

With the current data, we concluded that LND could not only improve the prognosis of selected ICC patients with clinically negative LNM, but also guide the postoperative management. However, patients intended to undergo LND should be prudently selected.

## Data Availability Statement

Publicly available datasets were analyzed in this study. These data can be found here: All data included in this study are available upon request by contact with the corresponding author (YZ, lamp197311@126.com).

## Ethics Statement

The studies involving human participants were reviewed and approved by the Mengchao Hepatobiliary Hospital of Fujian Medical University’s Ethics Committee (No. 2018_048_01). The patients/participants provided their written informed consent to participate in this study.

## Author Contributions

LW, JYL, SZ, XB, JMW, WG, FL, JW, YMZ, JDL, SC, and WZ offered the data. QK, LW, and ZL acquired, analyzed, and interpreted the data. QK and LW drafted the article. YYZ conceptualized and designed the study, critically revised the article, and gave the final approval. All authors contributed to the article and approved the submitted version.

## Funding

This study was supported by the Fujian Provincial Medical Center of Hepatobiliary, Science and Technology project of Fuzhou (Grant number: 2019-SZ-47).

## Conflict of Interest

The authors declare that the research was conducted in the absence of any commercial or financial relationships that could be construed as a potential conflict of interest.

The reviewer JL declared a shared affiliation, with no collaboration, with several of the authors QK, LW, ZL, YZ to the handling editor at the time of the review.

## Abbreviations

ICC, intrahepatic cholangiocarcinoma; LND, lymph node dissection; LNM, lymph node metastasis; pLNM, pathologically confirmed lymph node metastasis; PSM, propensity score matching; OS, overall survival; HR, hazard ratio; CI, confidence interval; ECOG, the Eastern Cooperative Oncology Group; MF, mass forming; MVI, microvascular invasion; p-AT, postoperative adjuvant therapy; TACE, transarterial chemoembolization; CT, computed tomography; MRI, magnetic resonance imaging.
